# Relatively lower-intensity physical activity during leisure time and presenteeism among Japanese workers

**DOI:** 10.1093/joccuh/uiaf037

**Published:** 2025-07-04

**Authors:** Koki Nagata, Shohei Yamamoto, Yosuke Inoue, Haruka Miyake, Hiroko Okazaki, Toshiaki Miyamoto, Takeshi Kochi, Isamu Kabe, Aki Tomizawa, Maki Konishi, Seitaro Dohi, Tetsuya Mizoue

**Affiliations:** Department of Epidemiology and Prevention, Center for Clinical Sciences, Japan Institute for Health Security, 1-21-1 Toyama, Shinjuku-ku, Tokyo 162-8655 Japan; Department of Epidemiology and Prevention, Center for Clinical Sciences, Japan Institute for Health Security, 1-21-1 Toyama, Shinjuku-ku, Tokyo 162-8655 Japan; Department of Epidemiology and Prevention, Center for Clinical Sciences, Japan Institute for Health Security, 1-21-1 Toyama, Shinjuku-ku, Tokyo 162-8655 Japan; Department of Epidemiology and Prevention, Center for Clinical Sciences, Japan Institute for Health Security, 1-21-1 Toyama, Shinjuku-ku, Tokyo 162-8655 Japan; Mitsui Chemicals, Inc, Tokyo, Japan; Nippon Steel Corporation, East Nippon Works, Kimitsu Area, Chiba, Japan; Furukawa Electric Co, Ltd, Tokyo, Japan; KUBOTA Corporation Co, Ltd, Ibaraki, Japan; Health Design Inc, Tokyo, Japan; Department of Epidemiology and Prevention, Center for Clinical Sciences, Japan Institute for Health Security, 1-21-1 Toyama, Shinjuku-ku, Tokyo 162-8655 Japan; Mitsui Chemicals, Inc, Tokyo, Japan; Department of Epidemiology and Prevention, Center for Clinical Sciences, Japan Institute for Health Security, 1-21-1 Toyama, Shinjuku-ku, Tokyo 162-8655 Japan

**Keywords:** exercise, physical activity, productivity, work performance, presenteeism, occupational health

## Abstract

**Objectives:**

This study examines the cross-sectional association between relatively lower-intensity physical activity (LIPA) during leisure time and presenteeism, accounting for relatively higher-intensity physical activity (HIPA) during leisure time.

**Methods:**

Data were derived from 11 438 workers from 6 worksites of large companies in Japan, which participated in a questionnaire survey conducted between fiscal years 2018 and 2020. Frequency and duration per occasion were assessed for leisure-time physical activities at 3 intensities as determined by shortness of breath. LIPA was defined as activity not causing shortness of breath. The participants were divided into 3 groups according to LIPA volume (none, <60 min/wk, or ≥60 min/wk) and into 2 groups according to HIPA volume (none or engaged). A single question assessed the participants’ presenteeism through self-ratings of their work performance. A multivariable Poisson regression model with a robust variance estimator estimated prevalence ratios (PRs) of presenteeism and their 95% CIs across the categories for both LIPA and HIPA.

**Results:**

The prevalence of presenteeism tended to decrease with increasing amounts of LIPA and HIPA (*P* for trend <.001). Among those who did not engage in HIPA, a significantly lower prevalence of presenteeism was observed among individuals who engaged in LIPA for ≥60 min/wk compared with those who did not (adjusted PR = 0.74; 95% CI = 0.68-0.81).

**Conclusions:**

This study supports the protective role of LIPA during leisure time that does not cause shortness of breath against presenteeism among workers who do not engage in HIPA during leisure time.

## Teaser

Key points:

- Relatively higher-intensity physical activity (HIPA) is known to help prevent presenteeism; however, the association of relatively lower-intensity physical activity (LIPA) during leisure time, which can be easily engaged in, with presenteeism has been unclear.
- The present study demonstrates that LIPA during leisure time was associated with a lower prevalence of presenteeism only among those who did not engage in HIPA. Moreover, a dose–response relationship suggested that approximately 180 min/wk of LIPA may be particularly effective for reducing presenteeism in such individuals.
- The findings support the promotion of LIPA as an occupational health strategy for preventing presenteeism in individuals with low levels of HIPA.

## 1. Background

Presenteeism, which refers to the reduced work performance of workers who attend work while ill,^1^ is a significant public health issue affecting both individuals and organizations.^2^^,^^3^ The financial burden caused by presenteeism is considerable, potentially reaching around 60% of health-related costs.^1^^,^^4^ As such, implementing strategies to prevent presenteeism is crucial for enhancing company productivity. Previous studies have identified obesity,^5^ alcohol consumption,^5^ smoking,^5^ and an unhealthy diet^5^^,^^6^ as modifiable risk factors for presenteeism.

Physical activity (PA) is another factor for preventing presenteeism.^7^ A scoping review of 57 studies, including cross-sectional, longitudinal, and interventional studies, published by 2020, found an inverse association between PA and presenteeism.^7^ A longitudinal study of 19 121 workers indicated that those who engage in adequate exercise—defined as engaging in exercise lasting for 30 minutes or longer on 3 days or more in the past 7 days—have a decreased risk of presenteeism 1 year later compared with those who engage in exercise inadequately.^8^ Another longitudinal study of 6515 workers examined the changes in relatively higher-intensity physical activity (HIPA) and presenteeism and found that increased HIPA was associated with decreased presenteeism.^9^

However, there are 3 unresolved issues in the previous studies on the association between PA and presenteeism. First, few attempts have been made to examine the association between relatively lower-intensity physical activity (LIPA) and presenteeism, except for Brown et al,^10^ who showed that the groups with a longer total duration of objectively measured low-intensity PA have a lower prevalence of presenteeism than the groups with a shorter duration. This is an important omission in knowledge, given that a growing body of research has suggested the possible benefits of low-intensity PA in relation to several health outcomes.^11^ Examining the association between LIPA during leisure time and presenteeism might inform efforts to maintain workplace productivity. Second, to accurately identify the association between LIPA and presenteeism, it is essential to adjust for HIPA, which is known to decrease the risk of presenteeism^8^^,^^9^; however, this topic has not been hitherto examined. Third, the relationship between LIPA and presenteeism in those who do not engage in HIPA is not known. From a public health perspective, it would be crucial to determine if presenteeism could be decreased by engaging only in LIPA, which is more easily performed than HIPA. In addition, the examination of dose–response relationships can reveal the duration of LIPA at which the prevalence of presenteeism is lowest. The findings can then be used to develop measures to prevent and improve presenteeism.

To address the above-mentioned issues, we examined the association between LIPA during leisure time and presenteeism after adjusting for HIPA. We also repeated the analysis by stratifying the HIPA level.

## 2. Methods

### 2.1. Study setting

This study is part of the Japan Epidemiology Collaboration on Occupational Health (J-ECOH) study,^12^^,^^13^ which collects health-related information (eg, annual health checkups, mortality, cardiovascular disease, long-term sickness absence) from employees of several large companies in various industries (eg, electrical machinery and apparatus manufacturing, steel, and chemicals) headquartered in the Kanto and Tokai regions. In the third phase of the J-ECOH study (fiscal years 2018–2020), we conducted a questionnaire survey at 6 worksites: paper-based questionnaires (4 worksites) or web-based questionnaires (2 worksites). We explained the purpose of the study to the employees participating in the present study on the first page or window of the questionnaire. Consent was obtained when a potential participant signed and returned the paper-based questionnaire or clicked the consent box of the web-based questionnaire. This study was conducted according to the guidelines of the Declaration of Helsinki, and the study protocol was approved by the Research Ethics Committee of the National Center for Global Health and Medicine (approval number: NCGM-S-001140).

### 2.2. Participants

**Figure 1:**
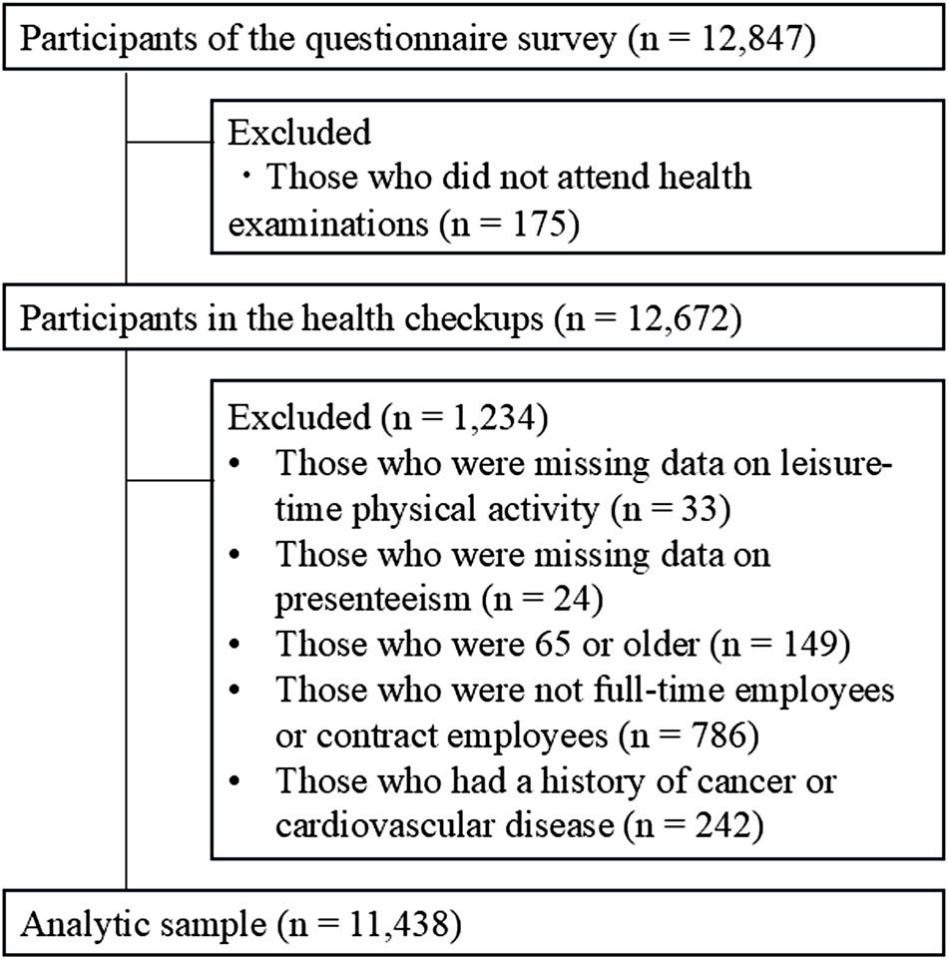
Flow diagram of the study participants.

Figure 1 shows the flow of the participants in this study. Of the 12 847 individuals who answered the questionnaire survey, 12 672 also took part in the annual health checkup. We excluded those who were missing data on leisure-time PA (*n* = 33) or presenteeism (*n* = 24), were aged 65 years or older (*n* = 149), were not full-time workers or contract workers (*n* = 786), and had a history of cancer or cardiovascular disease (*n* = 242), leaving 11 438 participants (10 044 males and 1394 females) to be included in the analysis.

### 2.3. Leisure-time PA

We developed our questionnaire on leisure-time PA by adapting items from established questionnaires.^14^^,^^15^ For each intensity of activity, that is, LIPA, moderate-intensity PA, and vigorous-intensity PA, we collected data on frequency and duration for each session. In the questionnaire, the intensity of PA was explained with the aid of illustrative examples. LIPA was defined as “activities of an intensity that does not cause shortness of breath” (eg, strolling, walking, calisthenics, yoga, golf, and gardening as a hobby). Moderate-intensity PA was defined as “activities of an intensity that causes shortness of breath but still allows conversation” (eg, light jogging, swimming, skiing, dancing, and light ball games). Vigorous-intensity PA was defined as “activities of an intensity that causes severe shortness of breath, making conversation difficult” (eg, martial arts, intense ball games, and marathon running). We assigned the values to each response option for frequency (none = 0; 1-3 times/mo = 0.5; 1-2 times/wk = 1.5; 3-4 times/wk = 3.5; 5-6 times/wk = 5.5; and almost daily = 7) and duration per session (<30 minutes = 15; 30-59 minutes = 45; 1.0-1.9 hours = 90; 2.0-2.9 hours = 150; 3.0-3.9 hours = 210; and ≥4.0 hours = 270). The activity duration per week was calculated by multiplying the frequency and duration per session for each intensity. For HIPA, the activity duration per week was calculated by summing the durations for moderate-intensity PA and vigorous-intensity PA.

We categorized participants into 3 groups for analyzing LIPA and HIPA: none, <60 min/wk, and ≥60 min/wk; the cutoff was close to the median LIPA (67.5 min/wk) and HIPA (75.0 min/wk). We then created a variable combining HIPA (none or engaged) and LIPA (none, <60 min/wk, or ≥60 min/wk) and divided participants into 6 groups.

### 2.4. Presenteeism

We assessed presenteeism using a single question: “If your ability (performance) at work when you are healthy is 100%, at what level did you perform on average over the last month?” This question was developed for our study, with reference to the Japanese version of the World Health Organization Health and Work Performance Questionnaire short form.^16^ Participants were asked to choose the closest number from a list of choices separated from 10% to 100% in increments of 10. A higher score indicated lower presenteeism (higher work performance). We defined presenteeism as a score of 60% or below. We have arbitrarily set this cutoff point so that the proportion of presenteeism among the study participants would approximate that reported in a study of the working population in Japan.^17^

### 2.5. Covariates

The following data on sociodemographic factors were collected through the questionnaires: age (<30, 30-39, 40-49, 50-59, or 60-64 years), sex (male or female), education (≤12, 13-16, or ≥17 years), marital status (married/cohabiting, never married, or separated/bereaved), job position (senior management/middle management or other), job type (management/supervision, professional/technical, research/development, clerical, sales, maintenance, production line, or other), work shift (day shift, shift work, flex, or other), overtime hours worked last month (0-10, 11-30, or ≥31 hours), and PA at work. Based on a previous study,^18^ the PA at work was categorized into 3 groups by combining the time spent standing at work and the time spent on heavy material handling: (1) sedentary work: no heavy material handling and standing for <2 h/d; (2) semi-sedentary work: standing for ≥2 h/d without heavy material handling or heavy material handling for <1 h/d regardless of standing hours; and (3) physically active work: heavy material handling for ≥1 h/d regardless of standing hours.

We also collected data on the following lifestyle-related factors: smoking (conventional cigarettes or heated tobacco products), alcohol consumption, sleep duration, and diet. Alcohol consumption was assessed based on consumption frequency (never drink, quit, 1-3 d/mo, 1-2 d/wk, 3-4 d/wk, 5-6 d/wk, or daily) and the amount consumed per occasion (0.5, 1, 1.5, 2, 2.5, 3, 3.5, or 4 *go* or more; a *go* is the traditional Japanese unit, equivalent to approximately 20 g of ethanol). We assigned values to each response option for consumption frequency (never drink/quit = 0; 1-3 d/mo = 0.5; 1-2 d/wk = 1.5; 3-4 d/wk = 3.5; 5-6 d/wk = 5.5; and daily = 7), which were then multiplied with the amount to calculate the daily alcohol consumption. Alcohol consumption was entered into the analysis using 4 categories: 0, 0.1-0.9, 1.0-1.9, or ≥2 *go*/wk. Sleep duration was assessed based on the time of going to bed and waking up on weekdays and weekends. The number of sleep hours on weekdays and weekends was calculated; the average sleep duration was also calculated using the following formula: (weekday sleep duration × 5 + weekend sleep duration × 2)/7. If the average sleep duration exceeded 16 hours, it was considered missing. The average sleep duration was entered into the analysis under 3 categories: <6.0, 6.0-6.9, or ≥7.0 hours. Diet was assessed using the Modified Japanese Diet Score—details given elsewhere.^19^ This diet score consists of 11 items, with 1 point awarded for each food consumed at the defined frequency and a higher score indicating a healthier diet. The total score was categorized into quartiles (≤2, 3-4, 5-6, or 7-11 points) for the analysis.

The body mass index (BMI) was calculated by measuring height and weight. The following 4 categories were used for the analysis: <18.5, 18.5-24.9, 25.0-29.9, or ≥30.0 kg/m^2^.

Participants were considered to have hypertension if their systolic blood pressure was ≥140 mmHg, diastolic blood pressure ≥90 mmHg, or they were taking antihypertensive medication. Diabetes was defined as having a fasting plasma glucose level ≥126 mg/dL, random glucose level ≥200 mg/dL, glycated hemoglobin (HbA1c) ≥6.5%, or using antidiabetic medication. Dyslipidemia was defined as having triglycerides ≥150 mg/dL, low-density lipoprotein cholesterol ≥140 mg/dL, high-density lipoprotein cholesterol <40 mg/dL for males and <50 mg/dL for females, or using antidyslipidemia medication.

### 2.6. Statistical analyses

The number of missing data points was as follows: education (*n* = 26), marital status (*n* = 22), job position (*n* = 5), job type (*n* = 13), overtime hours worked last month (*n* = 6), PA at work (*n* = 1), smoking (*n* = 3), alcohol consumption (*n* = 11), sleep duration (*n* = 21), diet score (*n* = 54), BMI (*n* = 1), and dyslipidemia (*n* = 89). Assuming that the data were missing at random, we performed multiple imputations using the chained equation method. We created 20 multiple imputation datasets that included all the measurement variables and combined the estimated parameters using the Rubin methods.^20^ To examine the association between each PA variable (HIPA, LIPA, and the combination of HIPA and LIPA) and presenteeism, we conducted a multilevel Poisson regression analysis using robust variance estimators and estimated the prevalence ratios (PRs) and 95% CIs for the outcome across exposure levels. Due to the differences in industrial sectors and survey timing across study worksites, we treated study sites as a random effect in all models to appropriately model between-worksite heterogeneity. We created 3 models: model 1 was unadjusted, model 2 was adjusted for age and sex, and model 3 was adjusted for age, sex, education, marital status, job position, job type, work shift, overtime hours worked last month, PA at work, smoking, alcohol consumption, sleep duration, diet score, BMI, hypertension, diabetes, dyslipidemia, and the other PA intensities during leisure time (eg, adjust for HIPA when examining LIPA and presenteeism). To investigate the dose–response relationship between LIPA and the prevalence of presenteeism stratified by HIPA (none or engaged), the model was run while treating LIPA (continuous) as a cubic polynomial (terms LIPA, LIPA^2^, and LIPA^3^). Data for those engaging in more than 330 minutes of LIPA during leisure time were truncated (3.83% of the distribution) due to the insufficient sample size for estimation. We repeated the above analysis stratified by sex and survey period (before COVID-19 [April 2018 to December 2019] or during COVID-19 [April 2020 to March 2021]). We evaluated the statistical interactions by adding the interaction terms of LIPA and each stratifying variable to the model. Stata/MP 18.0 (Stata Corp, College Station, TX, USA) was used for all statistical analyses. All tests were 2-sided, and *P* < .05 was considered statistically significant.

## 3. Results

The mean age of the participants was 40.9 years (SD = 11.5). Of all participants, 14.0% were classified as experiencing presenteeism. Table 1 depicts the characteristics of the participants according to the LIPA and HIPA levels. Among both the participants who engaged in HIPA and those who did not, those who engaged in LIPA for longer were more likely to be older, married, hold management positions, engage in clerical work, work fewer overtime hours, and have a higher Japanese diet score. Conversely, they were less likely to be current smokers. In the group who did not engage in HIPA, those who engaged in LIPA for more hours were more likely to have a higher level of education and to work in sedentary positions, but were less likely to be shift workers.

**Table 1:** Participant characteristics for relatively lower- and higher-intensity physical activity combinations (*n* = 11 438).^a^

	**HIPA: none**						**HIPA: engaged**					
	**LIPA min/wk**						**LIPA min/wk**					
	**None**		**<60**		**≥60**		**None**		**<60**		**≥60**	
**Number of participants**	2594		1930		1940		1033		1473		2468	
**Age, y**	40	[11.2]	42	[11.1]	45	[10.6]	37	[11.0]	39	[11.4]	41	[11.9]
**Male**	2234	(86.1)	1636	(84.8)	1630	(84.0)	975	(94.4)	1337	(90.8)	2232	(90.4)
**Education ≥17 y**	384	(14.8)	487	(25.2)	540	(27.8)	256	(24.8)	412	(28.0)	641	(26.0)
**Married**	1402	(54.1)	1273	(66.0)	1307	(67.4)	565	(54.7)	939	(63.8)	1634	(66.2)
**Management position**	532	(20.5)	670	(34.7)	817	(42.1)	274	(26.5)	508	(34.5)	955	(38.7)
**Job type**												
**Production line**	1231	(47.5)	569	(29.5)	484	(25.0)	367	(35.5)	514	(34.9)	767	(31.1)
**Clerical**	406	(15.7)	414	(21.5)	530	(27.3)	161	(15.6)	264	(17.9)	526	(21.3)
**Research and development**	341	(13.2)	371	(19.2)	342	(17.6)	185	(17.9)	271	(18.4)	419	(17.0)
**Other**	88	(3.4)	72	(3.7)	79	(4.1)	18	(1.7)	40	(2.7)	90	(3.7)
**Shift work**	700	(27.0)	325	(16.8)	226	(11.7)	215	(20.8)	334	(22.7)	465	(18.8)
**Overtime work ≥31 h/mo**	933	(36.0)	616	(31.9)	559	(28.8)	398	(38.5)	507	(34.4)	777	(31.5)
**Sedentary work**	1234	(47.6)	1224	(63.4)	1322	(68.1)	552	(53.4)	821	(55.7)	1463	(59.3)
**Current smoking**	937	(36.1)	541	(28.0)	513	(26.4)	285	(27.6)	338	(23.0)	533	(21.6)
**Alcohol consumption ≥2 *go*** ^**b**^ **/d**	275	(10.6)	190	(9.8)	208	(10.7)	90	(8.7)	113	(7.7)	234	(9.5)
**Sleep <6.0 h**	407	(15.7)	254	(13.2)	287	(14.8)	121	(11.7)	121	(8.2)	291	(11.8)
**Diet score 7-11 points**	264	(10.2)	302	(15.7)	403	(20.8)	145	(14.0)	272	(18.5)	585	(23.7)
**BMI ≥30.0 kg/m** ^**2**^	207	(8.0)	120	(6.2)	115	(5.9)	33	(3.2)	66	(4.5)	102	(4.1)
**Comorbid condition**												
**Hypertension**^**c**^	582	(22.4)	432	(22.4)	496	(25.6)	121	(11.7)	235	(16.0)	485	(19.7)
**Diabetes**^**d**^	119	(4.6)	104	(5.4)	131	(6.8)	18	(1.7)	48	(3.3)	91	(3.7)
**Dyslipidemia**^**e**^	1151	(44.4)	970	(50.3)	951	(49.0)	385	(37.3)	635	(43.1)	1067	(43.2)

Abbreviations: BMI, body mass index; HbA1c, glycated hemoglobin; HIPA, relatively higher-intensity physical activity; LIPA, relatively lower-intensity physical activity;

a Data are shown as means [SD] for continuous variables or numbers (%) for categorical variables.

b *go* is the traditional Japanese unit, equivalent to approximately 20 g of ethanol.

c Defined as systolic blood pressure ≥140 mmHg, diastolic blood pressure ≥90 mmHg, or taking antihypertensive medication.

d Defined as fasting plasma glucose level ≥126 mg/dL, random glucose level ≥200 mg/dL, HbA1c ≥6.5%, or using antidiabetic medication.

e Defined as triglycerides ≥150 mg/dL, low-density lipoprotein-cholesterol ≥140 mg/dL, high-density lipoprotein-cholesterol <40 mg/dL for males and <50 mg/dL for females, or using antidyslipidemia medication.

Table 2 presents the associations of HIPA and LIPA with the prevalence of presenteeism. HIPA was associated with a lower prevalence of presenteeism. The fully-adjusted PRs (95% CI) of presenteeism for none, <60 min/wk, and ≥60 min/wk of HIPA were 1.00 [reference], 0.86 (0.77-0.96), and 0.87 (0.83-0.91), respectively (*P* for trend <.001). LIPA was also associated with a lower prevalence of presenteeism. In model 3, PRs (95% CI) of presenteeism for none, <60 min/wk, and ≥60 min/wk were 1.00 [reference], 0.89 (0.75-1.05), and 0.83 (0.76-0.90), respectively (*P* for trend <.001).

**Table 2:** Multilevel Poisson regression results for the association between leisure-time physical activity and presenteeism.

			**Prevalence ratio (95% CI)**		
	**Case** ^**a**^ **/participants (%)**		**Model 1** ^**b**^	**Model 2** ^**c**^	**Model 3** ^**d**^
**HIPA**					
**None**	992/6464	(15.4)	Reference	Reference	Reference
**<60 min/wk**	250/2020	(12.4)	0.80 (0.73-0.89)	0.76 (0.68-0.85)	0.86 (0.77-0.96)
**≥60 min/wk**	365/2954	(12.4)	0.80 (0.76-0.85)	0.76 (0.72-0.82)	0.87 (0.83-0.91)
***P* for trend**			<.001	<.001	<.001
**LIPA**					
**None**	642/3627	(17.7)	Reference	Reference	Reference
**<60 min/wk**	458/3403	(13.5)	0.76 (0.63-0.93)	0.79 (0.66-0.94)	0.89 (0.75-1.05)
**≥60 min/wk**	507/4408	(11.5)	0.65 (0.56-0.76)	0.70 (0.62-0.78)	0.83 (0.76-0.90)
***P* for trend**			<.001	<.001	<.001

Abbreviations: HIPA, relatively higher-intensity physical activity; LIPA, relatively lower-intensity physical activity.

a Presenteeism was defined as reporting average work performance at or below 60% over the last month.

b Unadjusted.

c Adjusted for age and sex.

d Additionally adjusted for education, marital status, job position, job type, work shift, overtime hours worked last month, physical activity at work, smoking, alcohol consumption, sleep duration, diet score, body mass index, hypertension, diabetes, dyslipidemia, and physical activities of different intensities during leisure time (eg, adjust for HIPA when examining LIPA and presenteeism). The worksites were incorporated as a random effect in all models.

Table 3 shows the association between the combinations of LIPA and HIPA and presenteeism. Compared with the group with no HIPA and no LIPA, those who engaged in HIPA showed a lower prevalence of presenteeism, regardless of LIPA duration. The PRs (95% CI) of presenteeism for those who engaged in HIPA but had no LIPA, LIPA <60 min/wk, and LIPA ≥60 min/wk were 0.74 (0.69-0.79), 0.75 (0.65-0.86), and 0.73 (0.63-0.84), respectively, in model 3. Among individuals with no HIPA, higher LIPA levels were associated with a lower prevalence of presenteeism. Compared with the group with no HIPA and no LIPA, the PRs (95% CI) of those who engaged in no HIPA but engaged in LIPA <60 min/wk and those who engaged in no HIPA but engaged in LIPA ≥60 min/wk were 0.85 (0.70-1.02) and 0.74 (0.68-0.81), respectively, in model 3.

**Table 3:** Multilevel Poisson regression results for the association between combined relatively lower- and higher-intensity physical activity and presenteeism.

			**Prevalence ratio (95% CI)**		
	**Case** ^**a**^ **/participants (%)**		**Model 1** ^**b**^	**Model 2** ^**c**^	**Model 3** ^**d**^
**HIPA and LIPA**					
**No HIPA and no LIPA**	505/2594	(19.5)	Reference	Reference	Reference
**No HIPA and LIPA <60 min/wk**	270/1930	(14.0)	0.73 (0.57-0.92)	0.76 (0.61-0.94)	0.85 (0.70-1.02)
**No HIPA and LIPA ≥60 min/wk**	217/1940	(11.2)	0.58 (0.52-0.66)	0.64 (0.59-0.70)	0.74 (0.68-0.81)
**HIPA and no LIPA**	137/1033	(13.3)	0.69 (0.63-0.75)	0.65 (0.58-0.72)	0.74 (0.69-0.79)
**HIPA and LIPA <60 min/wk**	188/1473	(12.8)	0.65 (0.55-0.78)	0.64 (0.55-0.76)	0.75 (0.65-0.86)
**HIPA and LIPA ≥60 min/wk**	290/2468	(11.8)	0.61 (0.49-0.75)	0.62 (0.51-0.75)	0.73 (0.63-0.84)

Abbreviations: HIPA, relatively higher-intensity physical activity; LIPA, relatively lower-intensity physical activity.

a Presenteeism was defined as reporting average work performance at or below 60% over the last month.

b Unadjusted.

c Adjusted for age and sex.

d Additionally adjusted for education, marital status, job position, job type, work shift, overtime hours worked last month, physical activity at work, smoking, alcohol consumption, sleep duration, diet score, body mass index, hypertension, diabetes, and dyslipidemia. The worksites were incorporated as a random effect in all models.

**Figure 2:**
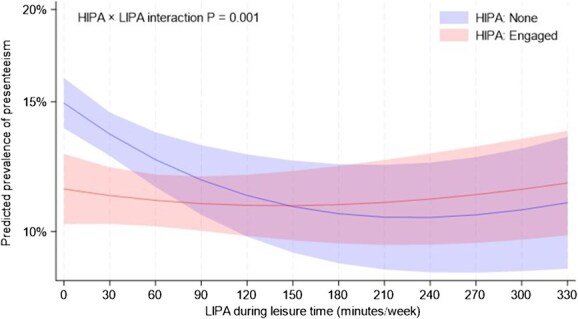
Dose–response association between relatively lower-intensity physical activity and presenteeism, stratified by relatively higher-intensity physical activity. The solid lines represent the predicted prevalence of presenteeism and the light-colored areas represent 95% CIs. Adjusted for age, sex, education, marital status, job position, job type, work shift, overtime hours worked last month, physical activity at work, smoking, alcohol consumption, sleep duration, diet score, body mass index, hypertension, diabetes, and dyslipidemia, with worksites incorporated as a random effect. HIPA, relatively higher-intensity physical activity; LIPA, relatively lower-intensity physical activity.

In Figure 2, the predicted prevalence of presenteeism in the group that did not engage in HIPA decreased with the increasing LIPA, leveling off at around 180 min/wk. By contrast, the group engaging in HIPA had a consistently low predicted prevalence of presenteeism, regardless of the LIPA duration (*P* for interaction = .001). There were no significant interactions by sex or survey period in the association between LIPA and presenteeism (Table S1).

## 4. Discussion

The present study showed that higher LIPA during leisure time that does not cause shortness of breath was associated with a lower prevalence of presenteeism among Japanese workers, even after adjustment for HIPA. Among those who did not engage in HIPA, the prevalence of presenteeism decreased with increasing LIPA up to approximately 180 min/wk, after which it plateaued.

The observed inverse association between LIPA and the prevalence of presenteeism is consistent with an Australian cross-sectional study showing a lower prevalence of presenteeism to be associated with longer durations of weekly and non-workday low-intensity PA.^10^ This may be explained by the favorable effects of LIPA on mental health and lower back pain, both of which are important determinants of presenteeism.^21^ Specifically, a longitudinal study of over 10 000 adults in Norway found that engaging in LIPA during leisure time was associated with a reduction in psychological distress.^22^ Moreover, meta-analyses have shown that lower-intensity exercises such as walking,^23^ stretching,^24^ and yoga^25^ are effective for the prevention and improvement of lower back pain. The present findings, together with those of previous studies, support the hypothesis that LIPA during leisure time can protect against presenteeism.

In the present study, among those who did not engage in HIPA, the prevalence of presenteeism decreased with increasing amounts of LIPA up to approximately 180 minutes of LIPA per week, suggesting a potential role of LIPA to reduce presentism among employees without HIPA. This finding is partially supported by an intervention study showing a reduction in presenteeism after a 30-minute lunchtime walking program among sedentary university employees (<150 min/wk of HIPA in any setting).^26^ Participants in the intervention study were encouraged to self-select their own walking intensity/speed for each walk. Thereafter, the researcher conducted a randomized controlled trial with a similar walking program under the same inclusion criteria, and found that the intervention group had increased enthusiasm and greater relaxation at work compared with the non-intervention group.^27^

Multidisciplinary approaches for both organizational and individual levels may effectively promote both HIPA and LIPA in leisure for workers. In the present study, long overtime work hours were associated with a lower likelihood of LIPA during leisure time, and similar findings have been reported in previous studies, suggesting that long working hours hinder leisure-time PA.^28^ Therefore, reducing overtime work may aid motivated workers to engage in or increase both HIPA and LIPA. The World Health Organization recommends replacing sedentary behavior with PA.^29^ For example, individuals can reduce the time spent watching TV or using a computer on holidays and instead go for a walk. In Japan, the “+10 min” initiative has been promoted to encourage individuals to increase their daily PA by 10 minutes.^30^ For instance, stretching exercises can be performed while watching TV. These initiatives may contribute to an increase in HIPA and LIPA during leisure time.

The strengths of our study include its large sample size of over 10 000 participants, the examination of the association between LIPA during leisure time and presenteeism while accounting for HIPA, and the investigation of the dose–response relationship to identify the LIPA duration associated with the lowest prevalence of presenteeism. Nevertheless, this study has the following limitations. First, we cannot infer a causal relationship between LIPA and presenteeism based on a cross-sectional study. Second, PA was self-reported using an unvalidated questionnaire developed for the J-ECOH study with reference to widely used questionnaires.^14^^,^^15^ In this questionnaire, exercise intensity was determined based on the degree of breathlessness. Therefore, our study’s classification of activity intensity may not fully align with classifications used in other standardized questionnaires, such as the International Physical Activity Questionnaire. Third, we have no information on the types of LIPA. Therefore, we cannot determine which LIPA is specifically associated with presenteeism. Fourth, presenteeism was also assessed using an unvalidated questionnaire developed for the J-ECOH study with reference to widely used questionnaires.^16^ Therefore, there is a possibility of misclassification in the assessment of presenteeism. Fifth, the possibility of bias due to residual confounding and unmeasured factors cannot be ruled out. For example, the heavy burden of childcare and caregiving is a known contributor to presenteeism^31^^,^^32^ and may limit opportunities for leisure-time PA due to time constraints. Consequently, participants engaged in substantial childcare or caregiving in our study might have exhibited lower levels of LIPA and a higher prevalence of presenteeism, potentially leading to an overestimation of the relationship between LIPA and presenteeism. Finally, the participants were predominantly young and middle-aged male employees in large companies in Japan. Therefore, caution should be taken when applying our findings to populations with different backgrounds.

## 5. Conclusions

This cross-sectional study of Japanese workers showed that LIPA during leisure time that does not cause shortness of breath was associated with a lower prevalence of presenteeism among individuals who do not engage in HIPA. The findings support the promotion of LIPA for individuals with low levels of HIPA as an occupational health strategy for the prevention of presenteeism. Longitudinal studies with objectively measured PA data are required to confirm the present cross-sectional associations.

## Supplementary Material

Web_Material_uiaf037

## Data Availability

The datasets used and/or analyzed during the current study are available from the corresponding author upon reasonable request.
