# Supplementary material for: Relatively lower-intensity physical activity during leisure time and presenteeism among Japanese workers
Source: J Occup Health. 2025 Jul 4;67(1):uiaf037. doi: 10.1093/joccuh/uiaf037 (PMC12303603; doi:10.1093/joccuh/uiaf037)
Supplement: Web_Material_uiaf037 [file web_material_uiaf037.zip › Supplementary_Material_R3.pdf]

Supplementary Table 1. Poisson regression results for the association between relatively lower-intensity physical activity during leisure time and presenteeism in the subgroups.

			Prevalence ratio (95% confidence interval)			P for trend
			None	< 60 minutes/week	≥ 60 minutes/week	
Sex	Male					
		Case[a]/participants (%)	584/3,209 (18.2)	410/2973 (13.8)	445/3,862 (11.5)	
		Model[bc]	Reference	0.88 (0.71–1.09)	0.80 (0.72–0.89)	< 0.001
	Female					
		Case[a]/participants (%)	58/418 (13.9)	48/430 (11.2)	62/546 (11.4)	
		Model[bc]	Reference	0.95 (0.69–1.31)	0.93 (0.75–1.16)	0.480
	P for interaction	Model[bc]	0.152			
Survey timing	Before COVID-19					
		Case[a]/participants (%)	306/1,857 (16.5)	188/1,257 (15.0)	193/1,626 (11.9)	
		Model[de]	Reference	1.05 (0.88–1.24)	0.88 (0.74–1.05)	0.170
	During COVID-19					
		Case[a]/participants (%)	336/1,770 (19.0)	270/2,146 (12.6)	314/2,782 (11.3)	
		Model[de]	Reference	0.78 (0.67–0.90)	0.78 (0.67–0.91)	0.001
	P for interaction	Model[de]	0.424			

[a] Presenteeism was defined as reporting average work performance at or below 60% over the last month.

[b] A multilevel Poisson regression analysis using robust variance estimators including worksites as a random effect.

[c] Adjusted for age, education, marital status, job position, job type, work shift, overtime hours worked last month, physical activity at work, smoking, alcohol consumption, sleep duration, diet score, body mass index, hypertension, diabetes, dyslipidemia, and relatively higher-intensity physical activity, with worksites incorporated as a random effect.

[d] A Poisson regression analysis using robust variance estimators.

[e] Adjusted for age, sex, education, marital status, job position, job type, work shift, overtime hours worked last month, physical activity at work, smoking, alcohol consumption, sleep duration, diet score, body mass index, hypertension, diabetes, dyslipidemia, and relatively higher-intensity physical activity.
